# Poly(vinyl alcohol)/Pullulan Composite Hydrogels as a Potential Platform for Wound Dressing Applications

**DOI:** 10.3390/gels9070580

**Published:** 2023-07-16

**Authors:** Ioana-Alexandra Plugariu, Maria Bercea, Luiza Madalina Gradinaru, Daniela Rusu, Alexandra Lupu

**Affiliations:** “Petru Poni” Institute of Macromolecular Chemistry, 41-A Grigore Ghica Voda Alley, 700487 Iasi, Romania; gradinaru.luiza@icmpp.ro (L.M.G.); rusu.daniela@icmpp.ro (D.R.); lupu.alexandra@icmpp.ro (A.L.)

**Keywords:** PVA, pullulan, hybrid hydrogel, viscoelastic behavior, swelling, drug delivery

## Abstract

Hydrogels are 3D networks with an excellent ability to retain a high amount of water or biological fluids, representing suitable candidates for wound dressing applications. They can provide a protective barrier and a moist environment, facilitating wound treatment. The present paper focuses on physical hydrogels obtained from poly(vinyl alcohol) (PVA) and pullulan (PULL) mixtures in different weight ratios by using the freezing/thawing method. Hybrid hydrogels of similar polymer compositions were prepared in the presence of 0.5% Laponite^®^ RD. The influence of polysaccharide and clay addition on the properties of PVA hydrogels was investigated. Scanning electron microscopy showed evidence of the inner porous structure. The viscoelastic properties were investigated in different shear conditions and revealed the influence of the hydrogel composition on the network strength. The swelling behavior was followed in physiological saline solutions at 37 °C and pH = 7.4. For all samples, a quasi-Fickian diffusion mechanism was found. The delivery of neomycin sulfate was studied in similar conditions as for the swelling tests (0.15 M NaCl solutions; 37 °C; pH = 7.4) and different kinetic models were used to determine the release mechanism. The Peppas–Sahlin approach described very well the in vitro drug release mechanism from the polymeric hydrogels in the absence of clay. However, the hybrid polymer/clay hydrogels showed the best fit with the Korsmeyer–Peppas model. According to the present study, the porous membranes containing 40–60% PULL (in absence of clay) are suitable for the release of therapeutic agents at wound sites in physiological conditions.

## 1. Introduction

Hydrogels represent water-insoluble networks with a high absorption capacity of water and biological fluids. A synergistic combination of various polymers, clays, proteins, and peptides generates composite hydrogels with different functions and improved properties. During recent years, multicomponent networks were obtained using synthetic [1,2] and natural [3,4] polymers and they were extensively investigated as potential materials for various biomedical applications [1,2,3,4,5]. The viscoelastic properties of hydrogels can be tuned during their preparation, resulting in characteristics similar to those of soft tissues. Generally, the hydrogels can be obtained using physical, chemical, or combined methods [5,6]. The physical hydrogels are promising drug delivery systems, with their high internal void fractions facilitating a high loading capacity and an effective control of the release of therapeutic agents [7,8].

Polysaccharides are found abundantly in nature, being widely used for preparing hydrogels [4,5]. Among them, pullulan (PULL) proved to be a versatile water-soluble natural polymer, produced by fungi (such as *Aureobasidium pullulans*), which forms viscous solutions, stable in time, over a wide range of temperatures and pH [9]. Due to its valuable properties (hydrophilicity, biocompatibility, high bioadhesion, biodegradability, non-toxicity, edibility, ability to form films that are oil-resistant and impermeable to oxygen, etc. [10]), it was accepted by the FDA and European Union as a safe biopolymer and has been used in various applications, such as food ingredients or packaging, pharmaceutical excipients for protein stabilization or drug delivery systems, cosmetics, wound healing, tissue engineering, and wastewater remediation [7,9,11].

On the other hand, poly(vinyl alcohol) (PVA) is a water-soluble synthetic polymer that presents a high ability to form hydrogels, either by physical or chemical crosslinking. The morphology, rheological, and mechanical properties can be tuned by selecting the system composition and the preparation conditions [12,13]. The hydrogels obtained by applying successive freezing/thawing cycles to PVA-containing solutions present a high interest due to the lack of toxicity (no crosslinking agent is used), high elasticity [14,15,16], and self-healing ability [16] of the resulting physical networks.

Despite the excellent properties of PULL or PVA, the combination of these two polymers in different materials is limited by their poor miscibility. Scanning electron micrographs and mechanical properties of PULL/PVA films prepared by the casting method from dimethyl sulfoxide solutions revealed the immiscibility of the two polymers over the entire composition range. The tensile strength and tensile modulus of the 40% PULL/60% PVA films were improved by crosslinking with glyoxal [17]. However, composite PVA/PULL hydrogels for tissue engineering applications were obtained by chemical crosslinking with sodium trimetaphosphate or by a combination of physical (by applying freezing/thawing cycles) and chemical crosslinking [18]. Interpenetrating PULL/PVA networks for the controlled delivery of pirfenidone were prepared using the water-in-oil emulsion crosslinking method in the presence of glutaraldehyde. The microspheres presented non-cytotoxicity and cell viability [19].

During the electrospinning of PVA/PULL aqueous solutions, an excess of PULL predominantly formed beads connected by very thin nanofibers, and an excess of PVA determined the formation of beaded nanofibers with an average diameter of 150–300 nm [20]. The water absorbency increased in the presence of clay [21,22]. PVA/PULL/zeolite L hybrid hydrogels were designed for drug delivery applications [23]. By using oxidized pullulan and PVA, the dynamic interactions between the functional groups induced the formation of hydrogels with self-healing behavior, able to recover the rest structure after they were subjected to large deformations [24].

Cefotaxime-sodium-loaded keratin/pullulan/PVA hydrogels showed an optimum oxygen permeability (above 8.2 mg/mL), vapor transmission rate of 1000 g/m^2^/day, adequate biocompatibility and mechanical strength, presenting a high potential as dressing membranes in diabetic wounds [25]. Also, hydrophilic hydrogels for wound dressings were obtained by the freeze–thawing of mixtures of PVA, PULL, poly-L-lysine, and gelatin. An in vitro hemolysis assay showed evidence of an improved biocompatibility and an increased adsorption of protein as compared to pure PVA hydrogels [26]. Thus, the combination of PULL with other natural and synthetic polymers increases the potential for various biomedical applications, such as tissue engineering, drug delivery systems, wound healing, or gene therapy [27]. In the presence of clay, the hydrogels deliver the enzymatic compounds and also change the environmental conditions of the wound site, improving the antibacterial activity [28].

The wounds represent damage of the skin tissues as a result of some external or internal stresses. Wound healing is a dynamic and complex process of reparation or the creation of new tissues, with the restoration of their functions. Wound dressings may present gas permeability, maintain a wet environment at the wound surface (acting as a barrier to bacteria), and remove the excess of exudates [29,30]. Various methods are used to treat wound infections, either using molecules or nanoparticles with inherent antimicrobial activity or hydrogels loaded with drugs or antimicrobial agents [31,32]. The inclusion of biomolecules into hydrogels designed as wound dressings is known as one of the most promising therapeutic approaches to protect the wounds and to accelerate the healing process [3,7,9,33,34]. Polysaccharides are combined with other organic or inorganic compounds to confer additional properties, such as improved mechanical [35] or therapeutic [36] properties.

There exists an increasing demand for wound dressings, and their appropriate selection reduces the healing time and the pain of the patient, improving the quality of the life [37]. The ability to incorporate a high amount of water or biological fluids into their network makes the hydrogels suitable candidates for wound dressings. They provide a moist environment and remove the excess wound fluid, thereby accelerating wound healing [3,9,33,34].

The present paper focuses on a combination of synthetic (PVA) and natural (PULL) polymers to obtain hydrogels by the freezing/thawing method. The effect of polysaccharide and clay addition on hydrogel properties and their ability to deliver an active substance were investigated and discussed.

## 2. Results and Discussion

Hydrogels containing different ratios of poly(vinyl alcohol) (PVA) and pullulan (PULL) were obtained from their aqueous solutions (5% wt.) by the freezing/thawing method. Samples of similar polymer compositions were prepared in the presence of 0.5% Laponite^®^ RD (clay concentration in aqueous media). Table 1 presents the polymer composition of hydrogel samples, except sample 10, which is a viscous fluid.

### 2.1. SEM Analysis

**Figure 1:**
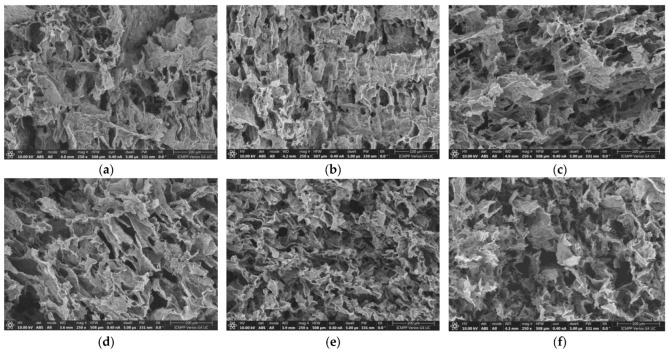
Scanning electron microscopy images of PVA/PULL hydrogel samples (Table 1): (**a**) 1, (**b**) 3, (**c**) 5, (**d**) 6, (**e**) 7, (**f**) 8.

**Figure 2:**
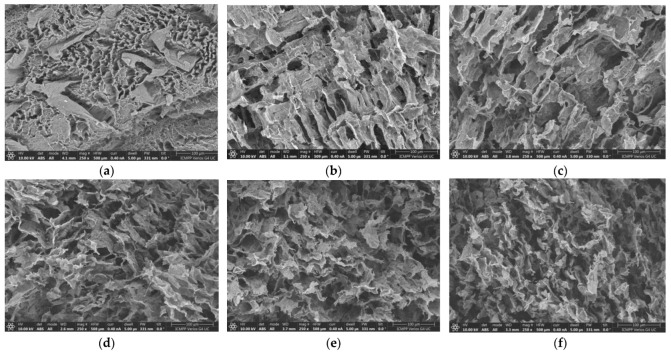
Scanning electron microscopy images of PVA/PULL hydrogel samples containing clay (Table 1): (**a**) 1C, (**b**) 3C, (**c**) 5C, (**d**) 6C, (**e**) 7C, (**f**) 8C.

Figure 1 shows SEM images for six selected hydrogels obtained in the absence of clay. A porous three-dimensional structure was observed for samples 1–8, while sample 9, with a low PVA content, appeared brittle due to the small number of crosslinking points. The network strength for this composition can be improved by subjecting the samples to a higher number of freezing/thawing cycles. According to rheological and SEM analysis, three cycles were selected for the preparation of the samples, ensuring the formation of the interconnected porous networks for polymer and hybrid hydrogels. Sample 10 did not form a network structure, regardless of the number of applied freezing/thawing cycles. The clay acts as a nanofiller; its addition to the polymer network can improve the rheological, thermal, and barrier properties [38,39], contributing to an extended wall formation, especially for high PVA content (Figure 2).

The analysis of SEM images shows that both types of hydrogels present a highly porous structure with interconnected pores. During freezing/thawing cycles, a phase separation accompanies the formation of junction points in the network; thus, the long PULL chains contribute to the pore connectivity and consolidation of the pore wall. Thus, for PULL contents between 40% and 60% (samples 5–7), the pore sizes are between 20 and 50 μm and they slowly decrease for higher PVA contents (more junction points are formed during freezing/thawing process). Generally, this porous structure provides a better drug and nutrient diffusion and metabolite exchange.

### 2.2. Viscoelastic Behavior of Hydrogels

Rheology is one of the most useful techniques to quantify the gel properties in correlation with their applications. Small-amplitude oscillatory shear investigations reveal the viscoelastic response and give information of the network structure.

**Figure 3:**
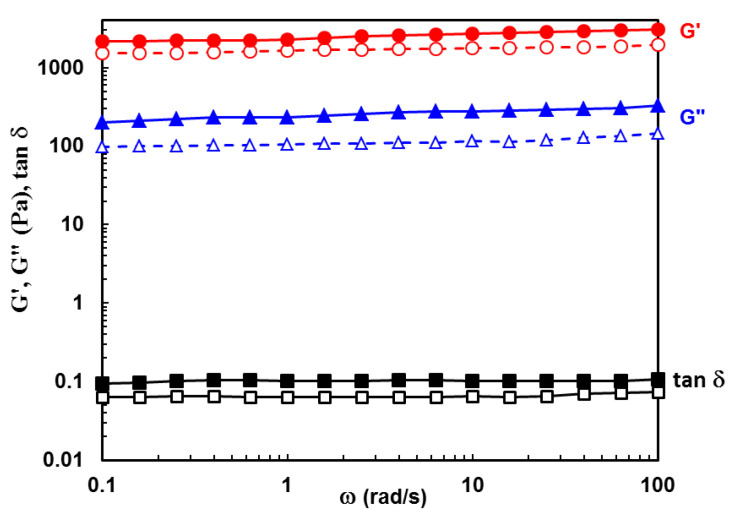
Viscoelastic parameters (G’, G”, and tan δ) as a function of oscillation frequency (ω) for PULL/PVA hydrogel with 10% PULL (sample 2, empty symbols) and the corresponding hybrid hydrogel (sample 2C, full symbols) at temperature of 37 °C.

Figure 3 shows the experimental data obtained in frequency sweep tests for PULL/PVA hydrogel with 10% PULL in the presence and absence of clay. G’ (elastic) and G” (viscous) moduli are nearly independent of oscillation frequency, with G’ > G”, and the loss tangent, tan δ = G”/G’, is around 0.1, indicating a network structure. For this composition, the clay addition slowly increases all viscoelastic parameters.

**Figure 4:**
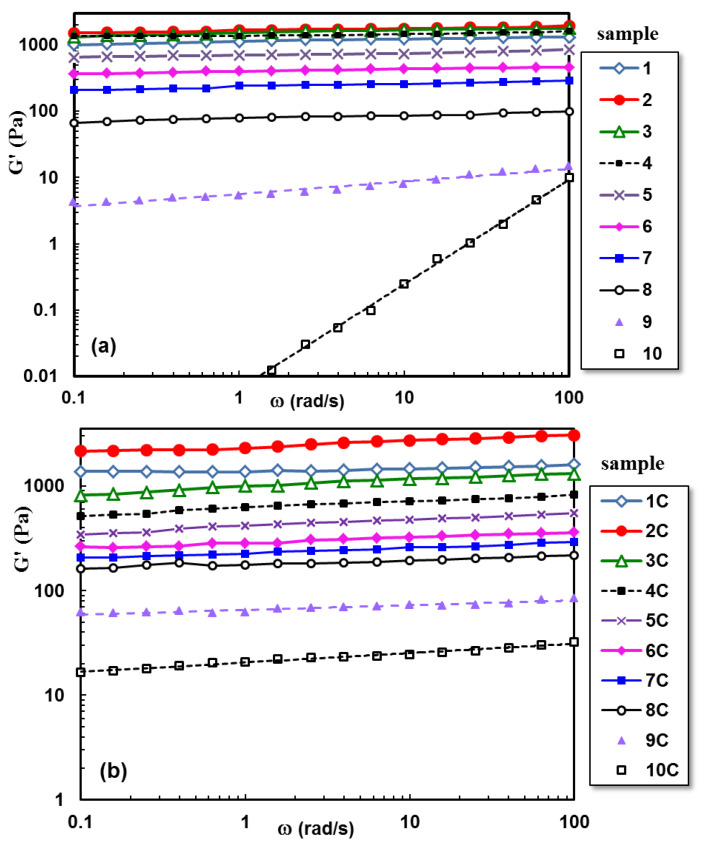
Elastic modulus as a function of PVA/PULL composition for (**a**) hydrogels without clay and (**b**) hybrid polymer/clay hydrogels, obtained by applying 3 freezing/thawing cycles (in the linear range of viscoelasticity, ω = 1 rad/s, γ = 1%, at temperature of 37 °C).

**Figure 5:**
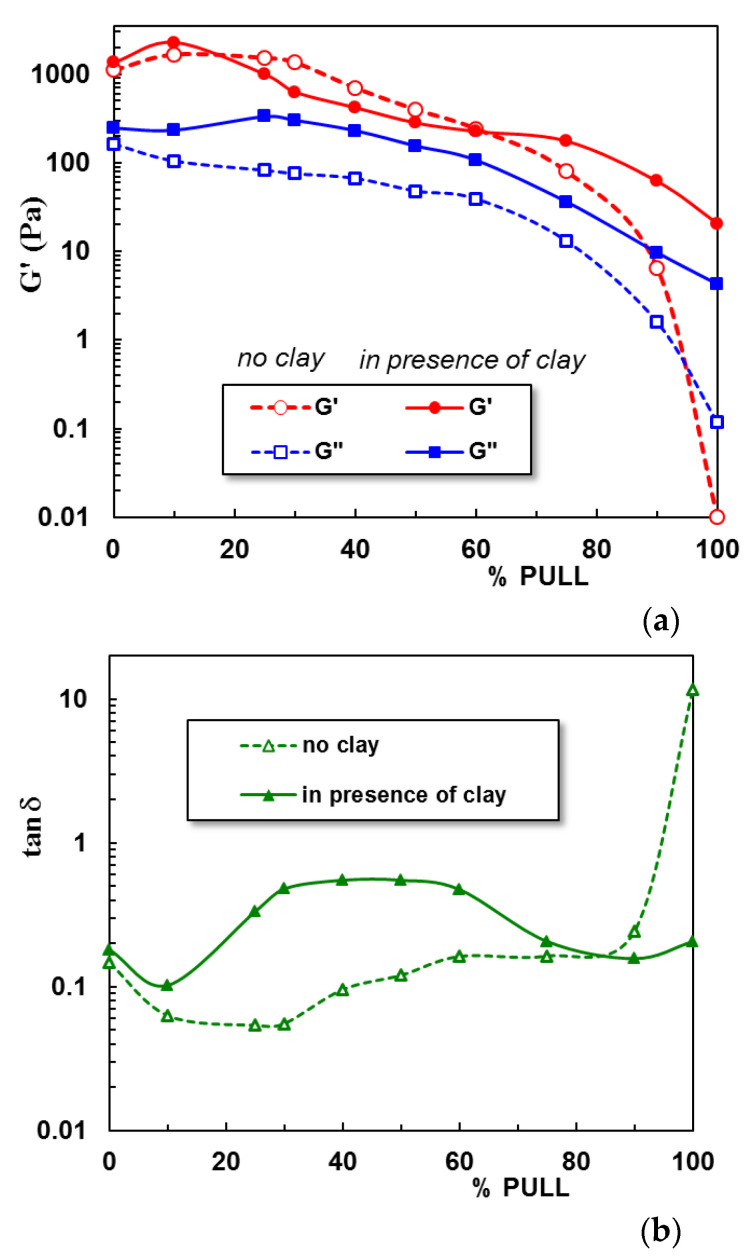
Elastic modulus and the loss tangent as a function of PVA/PULL composition for (**a**) hydrogels without clay and (**b**) hybrid polymer/clay hydrogels after 3 freezing thawing cycles (ω = 1 rad/s, γ = 1%, 37 °C).

The influence of polysaccharide and clay addition on the viscoelastic behavior of hydrogels is depicted in Figure 4 and Figure 5. Figure 4a presents the G’ dependence on the oscillation frequency for polymeric hydrogels with increasing PULL content, and Figure 4b shows the behavior of hybrid polymer/clay hydrogels at 37 °C.

A synergistic effect is observed for low PULL content (sample 2), when intermolecular interactions determine an increase in the network strength. By subjecting the PVA-entangled solution to successive freezing/thawing cycles, crystalline junction zones appear, acting as physical crosslinking points and generating the network structure [23]. The hydrogel formation is attributed to intermolecular hydrogen bonds achieved between the −OH groups of PVA chains, on the one hand, and between PVA and PULL macromolecules, on the other hand. Furthermore, the chain entanglements contribute to the network strength. Further PULL addition gradually decreases the G’ value due to the decrease in the number of junctions between PVA macromolecules. Sample 10, containing only PULL chains, behaves as a viscous fluid (no physical crosslinking after applying even a high number of freezing/thawing cycles). This demonstrates that PVA is responsible for 3D network formation.

In the presence of clay (Figure 4b), the PVA–PVA interactions are slowly perturbed, influencing the network formation. Thus, the loss tangent (tan δ) values increase for PULL contents between 20% and 60% (Figure 5). When the polymer mixture is mostly composed of PULL macromolecules, the clay addition contributes to the formation of junction points between PULL chains during mixing, and the weak network is preserved after applying freezing/thawing cycles, determining an increase in G’ value (tan δ decreases).

The rheological behavior of entangled polymer solutions is not significantly affected by the presence of Laponite^®^ RD, because the chain entanglements hinder the formation of the platelet structure [40]. However, the addition of clay to polysaccharide-based systems determines qualitative changes in the flow behavior and mechanical spectra [41,42]. It was also shown that the structural state of Laponite^®^ RD dispersions depends on clay concentration and ionic strength [38,43]. The manifestation of attractive and repulsive interactions between particles can lead to arrested states (either attractive gel or repulsive glass), generating a variety of rheological behaviors, from quasi-Newtonian to viscoelastic and shear thinning fluids [43].

### 2.3. Swelling Behavior

**Figure 6:**
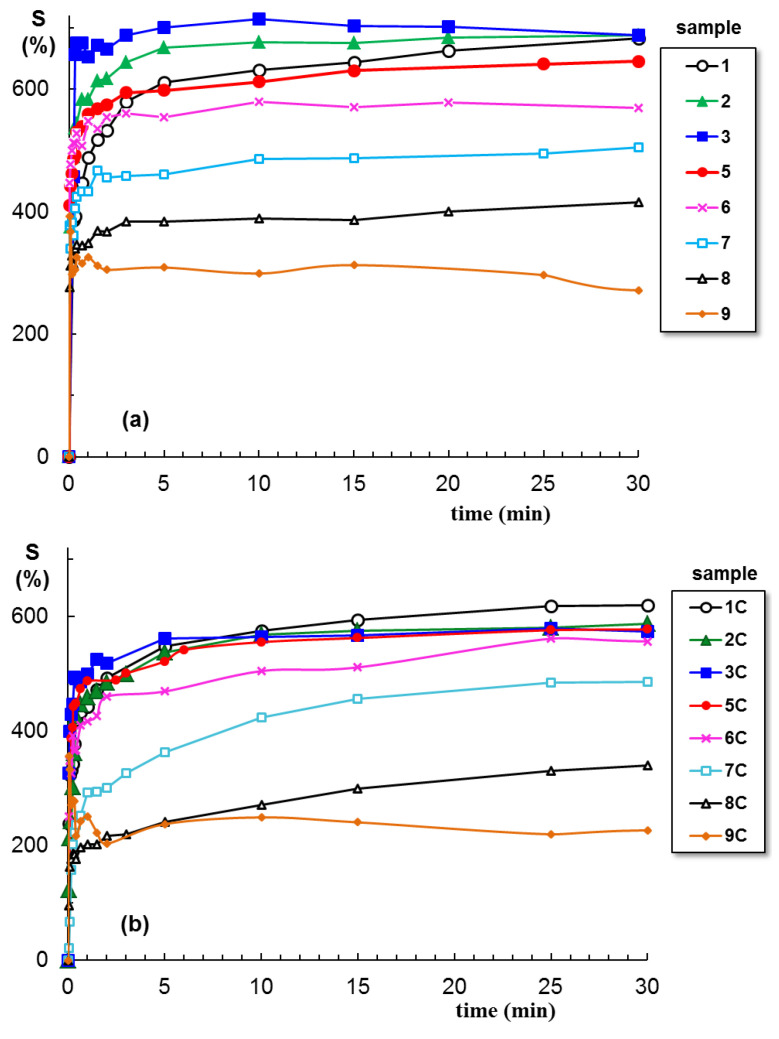
Swelling behavior of PVA/PULL hydrogels of different compositions (% wt. PULL) in physiological saline solutions at 37 °C and pH = 7.4.: (**a**) in absence of clay; (**b**) in presence of clay. The lines are a guide to the eye.

The swelling of hydrogels is carried out in NaCl solution at pH = 7.4. Figure 6 shows the swelling curves for hydrogels obtained in the absence and presence of clay. The first observation concerns the effect of clay: all polymer/Laponit samples present a smaller swelling degree as compared with similar polymer samples without clay. At equilibrium, a maximum swelling is obtained for sample 3 containing 25% PULL in the absence of clay, about 700% (Figure 6a), whereas PVA/clay hydrogel shows the maximum swelling level (around 600%, Figure 6b). A burst effect is registered for samples 9 and 9C, and the samples quickly disintegrate, with the network structure being unstable in the presence of the solvent.

**Figure 7:**
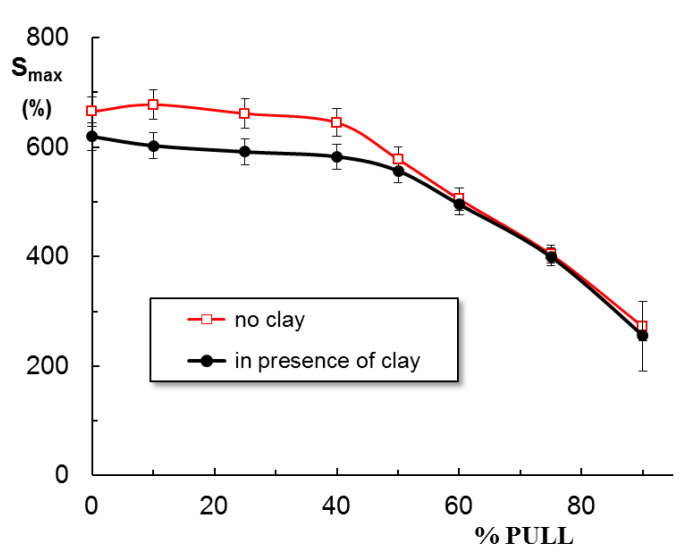
Swelling degree of hydrogels at equilibrium as a function of PVA/PULL mixture composition (% wt. PULL) in physiological saline solutions at 37 °C and pH = 7.4, in absence and in presence of clay.

The clay determines the decrease in swelling degree for samples 1 to 6 (Figure 7); then, when PULL chains are predominant (samples 7, 8, and 9), no significant differences are registered, in the limit of the experimental errors. The following relationship is used to determine the nature of the solvent diffusion through the hydrogel pores:

(1) $$F(t)=\frac{m_{t}}{m_{\infty }}=kt^{n_{s}}$$

where F(t) is the total water uptake at time *t*; $m_{t}$ and $m_{\infty }$ are the amounts of solvent absorbed by the network at a given time, *t*, and at equilibrium, respectively; *k* is a characteristic rate constant depending on the network structure; *n_s_* is a transport number indicating the solvent transport mode through the hydrogel pores (the swelling process could be controlled by diffusion and/or relaxation).

Equation (1) is applied to the early swelling stage, and from the slope of linear dependences of F(t) vs. *t*, the diffusion exponent ($n_{s}$) is obtained. For all PVA-containing hydrogels, $n_{s}$ < 0.5 (Table 1), suggesting a quasi-Fickian diffusion, when the initial diffusion of solvent molecules into the macroporous network occurs rapidly, and it is followed by a slow diffusion until the equilibrium state is reached. The deviations from Fickian diffusion are attributed to the interactions between the solvent molecules and the network constituents. It is found that the presence of clay or PULL in the PVA network has no influence on the diffusion mechanism.

Previously, by using water as a swelling environment, the following $n_{s}$ values were obtained: 0.18 for pure PVA hydrogel and 0.21 for PVA/Laponite^®^ RD hybrid hydrogel [38]. Furthermore, nanofibers of PULL, PVA, and montmorillonite, formed through the exfoliation of clay layers, are superabsorbent in distilled water (143.42 g/g), and the absorbency becomes much lower in 0.15 M NaCl solution (39.75 g/g) [44].

### 2.4. Neomycin Delivery

Neomycin is an antibiotic with bactericidal and bacteriostatic action, clinically tested against infections with *Trypanosoma cruzi* and Gram-positive bacteria (such as *Staphylococcus aureus)*, and very efficient against most Gram-negative bacterial strains (*Proteus vulgaris*, *Escherichia coli*, *Aerobacter aerogenes*, *P. aeruginosa*), without toxic effects [45,46].

**Figure 8:**
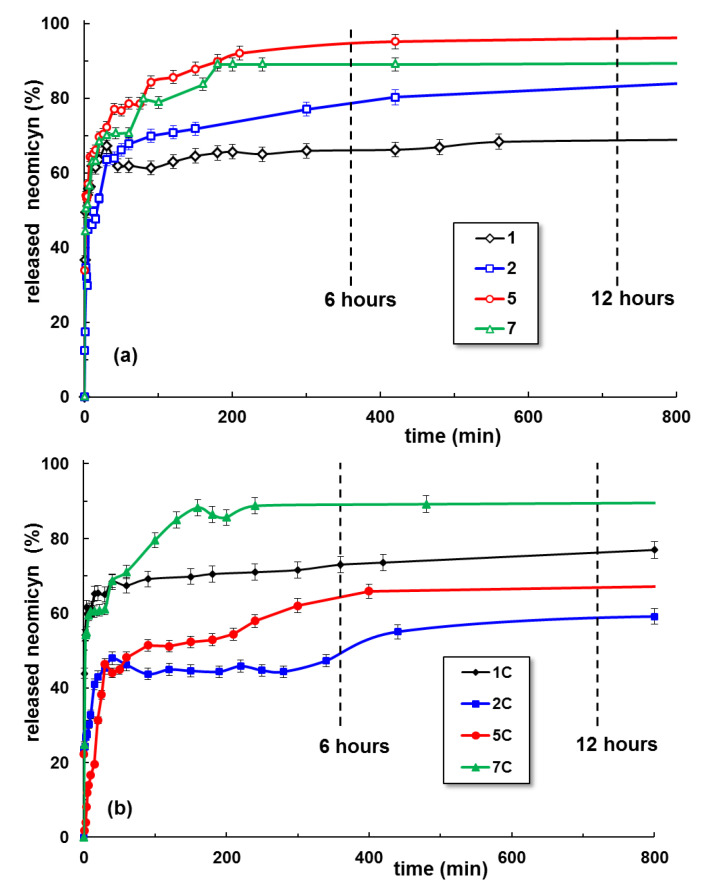
Neomycin delivery from hydrogels as a function of time, in physiological saline solutions, at 37 °C and pH = 7.4: (**a**) PVA/PULL hydrogels; (**b**) PVA/PULL/clay hydrogels. The lines are a guide to the eye.

Figure 8 presents the kinetics of neomycin release for some hydrogel samples selected on the basis of their swelling behavior shown in Figure 6 and Figure 7. The drug release begins when the hydrogel samples are introduced into the release medium and the small molecules of water and ions diffuse into the hydrogel. The mobility of polymer segments between two crosslinking points increases when the hydrogel swells, leading to a rise in drug mobility and determining its delivery in the environmental liquid. The maximum efficiency is obtained by the addition of 40–60% PULL to PVA hydrogels, whereas the clay improves the drug delivery for hydrogels with a high content of PULL.

The neomycin delivery from the hydrogels is discussed by using two models developed by Peppas and coworkers [47,48]:

Korsmeyer–Peppas [47]:

*M_t_*/*M*_∞_ = *k·t^n^* (2)

Peppas–Sahlin [48]:

*M_t_*/*M*_∞_ = *k*_1_·*t^m^* + *k*_2_·*t*^2*m*^ (3)

where *M_t_*/*M*_∞_ is the fraction of released drug at time *t*. The release kinetic constant *k* from Equation (2) depends on the characteristics of drugs [49]. Equation (2) is valid for the first drug release stages up to 60% of the maximum level. The release exponent *n* from Equation (2) is correlated with the release mechanism of the drug from the hydrogel (i.e., Fickian or non-Fickian diffusion). The constants *k*_1_ and *k*_2_ from Equation (3) also illustrate the Fickian or non-Fickian contributions to diffusion during the drug delivery [48]. For PVA/PULL hydrogels in the presence or absence of clay, *n* < 0.45 was obtained (Table 2), suggesting a pseudo-Fickian diffusion with slow drug release [47,50,51].

According to the present data, the Peppas–Sahlin approach (Equation (3)) [48] is more suitable to describe the in vitro drug release mechanism of neomycin from the polymeric hydrogels in the absence of clay (lower AIC values). The Korsmeyer–Peppas model [47] better describes the drug release from the PVA/PULL/clay hydrogels.

The release of neomycin, a water-soluble drug included in the matrix during the hydrogel’s preparation, is governed by the porous structure and the neomycin interactions with the network and the release environment. Each component of the network has a specific role, with a direct effect on rheological properties, swelling behavior, and drug release rate. PVA ensures the network formation after three freezing/thawing cycles due to the intermolecular hydrogen bonds in the crystalline regions acting as junction points in the network. When PVA macromolecules represent the majority, more crystalline regions are formed by applying successive freezing/thawing cycles, and this could be a detrimental parameter for drug release. The network is less extended by swelling in salted solutions and the amorphous regions have a limited diffusivity with consequences on the drug release kinetics. The occurrence of the burst effect observed for sample 1 can be correlated with a highly porous macrostructure of the PVA hydrogel (influenced by the crosslinking degree), drug diffusion through the pores in balance with the hydrogel swelling when the release begins, osmotic pressure in the diffusion membrane [52,53,54], or high drug loading (when some molecules are adsorbed or weakly bound to the hydrogel surface [38,55]). In pharmaceutical formulations, the burst effect should be avoided because it decreases the therapeutic response and the drug is lost in an uncontrolled and unpredictable manner [52,53,54]. For the physical crosslinked hydrogels, the use of a minimum PVA concentration (when the macromolecular chains are in entangled state [23]) is recommended for hydrogel preparation. For chemically crosslinked PVA hydrogels, the burst effect can be minimized by surface extraction or surface preferential crosslinking [52].

Due to the high hydrophilicity of PVA, its hydrogels have a weak ability to load the drugs and to avoid the initial burst effect [54]. In our study, the clay or polysaccharide addition improves the delivery during the first stage. The pullulan chains ensure the connectivity between different PVA crystalline and amorphous regions. By increasing the content of polysaccharide, the amorphous part of the hydrogel is extended and the overall mobility and diffusivity become significantly higher, contributing to a higher release rate. In addition, the very favorable interactions established between Na^+^ ions from the solvent and the monomeric units of PULL [56] improve the neomycin release. However, for polymer networks in the presence of clay, the increase in the ionic strength of the environment determines a diminution of the swelling degree and drug delivery ability from the hybrid gel matrix. 

## 3. Conclusions

Hydrogels containing a synthetic polymer (PVA) and a polysaccharide (PULL) in different ratios in the presence and absence of clay are prepared and their main structural and rheological characteristics are investigated. The swelling in physiological conditions (0.15 M NaCl solution, 37 °C) reveals a higher swelling degree in the absence of clay, favoring the drug release.

The models developed by Peppas and coworkers provide an accurate description of neomycin delivery. The Peppas–Sahlin approach describes the in vitro drug release mechanism from the PVA/PULL hydrogels in the absence of clay, whereas the Korsmeyer–Peppas model is more suitable for PVA/PULL clay networks. The introduction of PULL into the PVA physical network improves the hydrogel biocompatibility, but also determines the increase in the mobility and diffusivity in the amorphous zone, favoring the drug release. On the other hand, the clay addition is not beneficial, because as the ionic strength of the environment increases, the swelling is diminished and less drug is released from the network.

The PVA/PULL physical hydrogels could be considered effective carriers for antibiotics delivery, suitable for wound dressing applications. These porous hydrogels provide a wet environment in physiological conditions, and by absorbing the wound exudates, they reduce the risk of infection.

## 4. Materials and Methods

### 4.1. Materials

**Figure 9:**
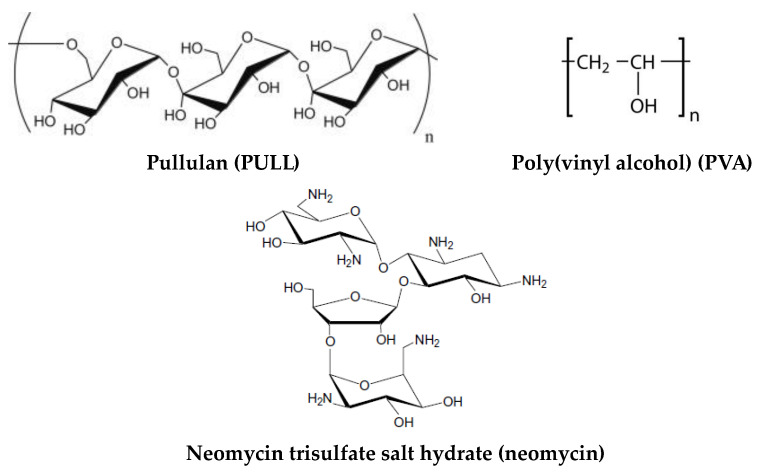
The chemical structure of polymers and used drug.

Poly(vinyl alcohol) (PVA) with a molecular weight of 1.3 × 10^5^ g/mol (99% hydrolyzed) was purchased from Sigma-Aldrich (Taufkirchen, Germany) and used as received. Pullulan (PULL) sample of 3 × 10^5^ g/mol was purchased from TCI Europe N.V. An aminoglycoside antibiotic, neomycin trisulfate salt hydrate (denoted as neomycin), was purchased from Sigma-Aldrich (Taufkirchen, Germany). It is water-soluble (50 mg/mL) and enhances the cationic-lipid-mediated transfection of reporter plasmids and oligonucleotides [57]. The chemical structure of polymers and neomycin is given in Figure 9. Laponite^®^ RD with the chemical formula Na^+0.7^[(Si_8_Mg_5.5_Li_0.3_)O_20_(OH)_4_]^−0.7^ was provided by BYK Additives Ltd. (Widnes, UK).

### 4.2. Preparation of the Composite Hydrogels

PVA and PULL were dissolved in Millipore water for obtaining stock solutions of 5% wt. concentration (pH = 7.4). PVA solution was prepared under magnetic stirring at a temperature of about 90 °C and the homogeneous solution was kept for a few hours at room temperature. PULL was dissolved in Millipore water by shaking with a rolling mixer at room temperature, then the solution was stored in a refrigerator. PULL/PVA solutions of different compositions (Table 1) were prepared and homogenized under magnetic stirring. Solutions of similar compositions were mixed with clay under high-speed stirring, then ultrasonicated and used immediately to prevent the formation of aggregates.

The freezing of freshly prepared mixtures was performed in liquid nitrogen to avoid phase separation. The thawing step was performed at room temperature for 10 h. The gel formation was checked by rheological measurements and it was established that the application of 3 freezing/thawing cycles was optimum to obtain networks with a porous structure for drug delivery applications.

### 4.3. Scanning Electron Microscopy Studies

The morphology of hydrogels was examined on a cross-section of freeze-dried samples by using the Verios G4 UC Scanning Electron Microscope (Thermo Scientific, Brno, Czech Republic), operating at 10 kV in high-vacuum mode with a backscatter electron detector, ABS (Angular Backscattered Detector). The samples were coated with a 6 nm platinum layer using a Leica EM ACE200 Sputter coater prior examination to improve electrical conductivity and prevent charge buildup. The SEM images were analyzed at various magnifications. Image J software was used to determine the average pore sizes from the SEM micrographs and each value was obtained as an average of at least 80 dimensions.

### 4.4. Rheological Investigations

Rheological measurements were carried out with an MCR 302 Anton-Paar rheometer (Graz, Austria) by using a plane–plane geometry (the upper plate of 50 mm, gap of 500 μm) and a Peltier device for temperature control. A preliminary rheological study was performed for solutions of PVA/PULL mixtures of various concentrations in order to select the concentration for which the polymer chains are in an entangled state. Thus, for For the present study, the chosen polymer concentration was of 5% (wt.).

The viscoelastic behavior of hydrogels with various compositions was investigated at 37 °C in frequency sweep tests, in the linear range of viscoelasticity (established in amplitude sweep tests). The viscoelastic moduli (G’ and G”) and the loss tangent (tan*δ*) were determined for oscillation frequencies (ω) between 0.1 rad/s and 100 rad/s (at constant deformation, γ, of 1%). The loss tangent was determined as G”/G’ ratio and gives information about the degree of viscoelasticity of the sample.

### 4.5. Swelling Behavior

Saline solutions with concentrations similar to physiological serum (0.9 g/dL aqueous solution of NaCl) were used for swelling tests. The hydrogel swelling was studied at 37 °C and pH = 7.4. The dry hydrogel (weight *m_o_*) was immersed in saline solution and the weight of the swollen sample was determined (*m_t_*) at different times *t*. The swelling degree (*S*), as the ratio between the weight of the liquid in the swollen state at a given time (*m_t_*) and the weight of the dried sample (*m_o_*), was calculated using the following equation:

(4) $$s=\frac{m_{t}-m_{o}}{m_{o}}\cdot 100(\%)$$

### 4.6. In Vitro Drug Release Study

Samples with different compositions were loaded with drug (0.5% wt. neomycin sulfate) in the solution state. Then, they were subjected to freezing/thawing following the sample procedure mentioned above. In this way, the entire neomycin sulfate molecules were entrapped in the network. The in vitro drug release behavior was investigated by immersing each hydrogel sample in a closed bottle with 10 mL of saline solution (0.9 g/dL aqueous solution of NaCl, pH = 7.4) at 37 °C in a thermostatic chamber. At different time intervals, 1 mL of the release medium was withdrawn and replaced by an equal volume of fresh, pre-heated solvent, maintaining a constant volume. The amount of drug in the release medium was determined by UV spectrophotometry, using a standard calibration curve (R^2^ = 0.9924). The absorbance was recorded at 202 nm using a Libra UV-Vis spectrophotometer (Biochrom, Cambridge, UK).

The drug delivery results were presented as the mean of three values with standard deviation (±SD). The experimental data were analyzed using OriginPro 8.5 software to generate the best regression fits. The minimum values of the residual sum of the squares (*RSS*) were considered. The Akaike Information Criterion (AIC) [58] was used for the statistical analysis, being independent of the number of parameters (*p*) introduced by the model:

AIC = *N* ln(*SSR*) + 2 *p* (5)

where *N* represents the number of experimental data samples.

The smallest AIC values were used to consider the best-fitting approach for describing the drug release mechanism [7,59].

## Figures and Tables

**Table 1:** The composition of mixtures used for preparing hydrogels and the loss tangent obtained for the investigated samples after they were subjected to freezing/thawing cycles (strain of 1%, oscillation frequency of 1 rad/s, temperature of 37 °C). The values of the swelling exponent ($n_{s}$, Equation (1)) are also given.

Sample Code	PVA (% wt)	PULL (% wt.)	Clay (% wt.)	tan δ	$n_{s}$
1	100	0	0	0.1465	0.1002
2	90	10	0	0.0629	0.1137
3	75	25	0	0.0539	0.0822
4	70	30	0	0.0555	0.0937
5	60	40	0	0.0956	0.0811
6	50	50	0	0.1203	0.0521
7	40	60	0	0.1624	0.0882
8	25	75	0	0.1634	0.0349
9	10	90	0	0.2435	0.0258
10	0	100	0	11.7000	-
1C	100	0	0.5	0.1809	0.218
2C	90	10	0.5	0.1022	0.1894
3C	75	25	0.5	0.3330	0.1454
4C	70	30	0.5	0.4794	0.1297
5C	60	40	0.5	0.5500	0.1012
6C	50	50	0.5	0.5495	0.1210
7C	40	60	0.5	0.4735	0.1993
8C	25	75	0.5	0.2063	0.4367
9C	10	90	0.5	0.1568	0.4129
10C	0	100	0.5	0.2063	-

**Table 2:** The parameters that describe the kinetics of neomycin release from the hydrogels according to Equations (2) and (3) (saline solution, 37 °C, pH = 7.4).

Sample Code	Korsmeyer–Peppas Equation (2)		Peppas–Sahlin Equation (3)			
	*n*	AIC	*k*_1_ (min^−m^)	*k*_2_ (min^−2m^)	m	AIC
1	0.1339	−79.2907	−0.1481	0.3787	0.1602	−77.3351
2	0.3161	−32.5148	−3.2568	1.9673	0.2034	−68.2583
5	0.1661	−50.1921	−3.7674	2.3748	0.1216	−62.5688
7	0.2021	−69.0031	−3.3078	2.0284	0.1805	−62.8957
1C	0.0873	−82.4993	−0.0585	0.1397	0.1712	−71.4268
2C	0.1335	−73.3387	−4.9473	3.1669	0.1069	−67.7979
5C	0.2089	−60.4088	−2.7178	1.6331	0.2459	−56.6507
7C	0.1788	−70.5489	−2.6381	0.5584	0.2036	−57.4339

## Data Availability

Data are available on request.
